# Peripheral blood CD19 positive B lymphocytes increase after ischemic stroke and correlate with carotid atherosclerosis

**DOI:** 10.3389/fneur.2023.1308041

**Published:** 2023-12-29

**Authors:** Yuhua Zhang, Yu Jiang, Yutian Zou, Yinyin Fan, Ping Feng, Xiang Fu, Keru Li, Jinru Zhang, Yunlei Dong, Shuying Yan, Yanlin Zhang

**Affiliations:** ^1^Department of Neurology and Clinical Research Center of Neurological Disease, The Second Affiliated Hospital of Soochow University, Suzhou, China; ^2^Department of Neurology, Afflliated Changshu Hospital of Nantong University, Changshu, China; ^3^Department of Clinical Laboratory, The Second Affiliated Hospital of Soochow University, Suzhou, China

**Keywords:** B lymphocytes, CD19, lipid metabolism, carotid atherosclerosis, ischemic stroke

## Abstract

**Introduction:**

Atherosclerosis is the primary pathological basis of ischemic stroke, and dyslipidemia is one of its major etiological factors. Acute ischemic stroke patients exhibit imbalances in lymphocyte subpopulations, yet the correlation between these dynamic changes in lymphocyte subpopulations and lipid metabolism disorders, as well as carotid atherosclerosis in stroke patients remains poorly understood.

**Methods:**

We retrospectively analyzed the demographic data, risk factors of cerebrovascular disease, laboratory examination (lymphocyte subsets, lipid indexes, etc.), clinical features and c;/]-sity from December 2017 to September 2019 and non-stroke patients with dizziness/vertigo during the same period.

**Results:**

The results showed that peripheral B lymphocyte proportions are elevated in acute ischemic stroke patients compared with those of the control group (13.6 ± 5.3 vs. 11.7 ± 4.4%, *p* = 0.006). Higher B lymphocyte proportions are associated with concurrent dyslipidemia, increased levels of vascular risk factors including triglycerides (TG), total cholesterol (TC), low-density lipoprotein cholesterol (LDL-C), and very-low-density lipoprotein cholesterol (VLDL-C), as well as decreased levels of the protective factor high-density lipoprotein cholesterol (HDL-C). Elevated B lymphocyte proportions are independently correlated with carotid atherosclerosis in stroke patients.

**Discussion:**

We found CD19 positive B Lymphocytes increase after ischemic stroke and correlate with Carotid Atherosclerosis. Lymphocyte subpopulations should be highlighted in stroke patients.

## 1 Introduction

Globally, stroke remained the second-leading cause of death and the third-leading cause of death and disability combined in 2019. Ischemic stroke constituted 62·4% of all incident strokes. Its high incidence, mortality, and disability rates impose a significant burden on families and society (1, 2). Ischemic stroke has many risk factors, with atherosclerosis being its primary pathological basis.

Timely and effective treatment within the time window is the key to preventing the progression of ischemic stroke and improving its prognosis. Currently, acute-phase treatments for ischemic stroke primarily include ultra-early mechanical thrombectomy and intravenous thrombolysis (3), which benefit only a minority (<10%) of patients and may lead to hemorrhagic transformation (HT), potentially causing early deterioration in neurological function, early mortality, and poor prognosis (4). Furthermore, even in patients achieving vascular recanalization, 35% of them cannot restore effective perfusion and neurological function (5). The development of ischemic penumbra imaging techniques has made it an attractive and important strategy in the acute phase to salvage the ischemic penumbra and improve stroke outcomes (6). Research suggests that ischemic stress can activate immune cells, trigger inflammation, and programmatic cell death, playing a crucial role in the expansion of the ischemic core into the penumbral zone. Targeted immunomodulation is expected to improve outcomes in patients with ischemic stroke by salvaging ischemic penumbral tissue. S1P (Sphingosine 1-phosphate) signaling coordinates vascular functions in other organs, and S1P1 modulators including fingolimod show promise for the treatment of ischemic and hemorrhagic stroke. S1P coordinates lymphocyte trafficking, and lymphocytes are currently viewed as the principal therapeutic target for S1P1 modulation in stroke (7, 8). Professor Shi Fudong’s team in China discovered in animal models that the lymphocyte modulator fingolimod can reduce infarct size, improve collateral circulation, and enhance blood–brain barrier integrity (9–11). Dr. Francisco Campos’ team at Massachusetts General Hospital found in a mouse middle cerebral artery ischemia model that fingolimod treatment can alleviate ischemia-induced neurofunctional deficits, reduce infarct size, improve the neurofunctional outcome of thrombolysis therapy, and decrease the risk of hemorrhagic transformation when used in combination with t-PA. In patients with acute and anterior cerebral circulation occlusion stroke, oral fingolimod within 72 h of disease onset was safe, limited secondary tissue injury from baseline to 7 d, decreased microvascular permeability, attenuated neurological deficits, and promoted recovery. Extending the t-PA treatment window to 72 h (12, 13) suggests that targeted modulation of lymphocyte subpopulations in combination with thrombectomy/thrombolysis therapy holds promise as a prospective treatment strategy in the acute phase of ischemic stroke.

B Lymphocyte subpopulations play crucial regulatory roles by generating germinal centers, producing antibodies, and cytokines during both the acute and recovery phases of ischemic stroke (14). Atherosclerosis is the main pathological basis of ischemic stroke, and lipid metabolism disorders are the core link in the occurrence and progression of atherosclerosis. Monoclonal antibodies against B cell surface molecules (e.g., CD20) and survival factors (e.g., BAFF) have been shown to have a protective effect in atherosclerotic animal models (15–17), and targeting depletion of the B-cell surface co-stimulatory molecules CD80 and CD86 can slow the development of atherosclerosis (18).

Different B lymphocytes play different roles in the different stage of stroke. In the acute stage of stroke, using an anti-CD20 antibody Pharmacologic depletion of B cells, lack of circulating B cells in JHD−/− mice or reconstitution of Rag1−/− mice with B cells did not influence infarct volumes and functional outcome at day 1 and 3 after stroke (19), but cell adoptive transfer to mice reduced infarct volumes 3 and 7 d after transient middle cerebral artery occlusion. B cell depletion by rituximab reduced stroke-induced hippocampal neurogenesis and cell survival (20), IL-10-producing B-cells limit CNS inflammation and infarct volume in experimental stroke (21, 22). Whole-brain volumetric serial two-photon tomography (STPT) and a custom-developed image analysis pipeline visualized and quantified poststroke B cell diapedesis throughout the brain showed that B cells migrate into remote brain areas regulating motor and cognitive functions and support neurogenesis and functional recovery after focal stroke in mice (20). However, B lymphocytes are involved in the development of post-ischemic stroke cognitive impairment by producing antibodies, μ MT (B-cell deletion) mice do not have delayed cognitive deficits, and the B-cell-targeted drug rituximab can reduce post-stroke cognitive impairment (23, 24).

These foundation show that targeted B-cell therapy has the potential to improve the prognosis of patients by regulating atherosclerosis, reducing thrombolytic hemorrhagic transformation, prolonging the window period of thrombolytic therapy, alleviating cognitive impairment after stroke, and participating in the whole process of ischemic stroke. However, the dynamics of lymphocyte subsets after ischemia and the key lymphocyte subsets that regulate ischemic brain tissue are unclear. This study aims to analyze the changes of peripheral blood lymphocyte subsets in patients with acute ischemic stroke, focusing on the correlation between B-cell subsets and lipid metabolism disorders as well as carotid atherosclerosis, the key risk factors of stroke. In order to provide a reference for the promotion of immunointerventional therapy for ischemic stroke.

## 2 Materials and methods

### 2.1 General materials

Retrospective data collection was conducted on acute ischemic stroke patients admitted to the Department of Neurology, Second Affiliated Hospital of Soochow University, from December 2017 to September 2019. This study was approved by the Ethics Committee of the Second Affiliated Hospital of Soochow University (EC-AF(SQ)-12/20210601). Inclusion Criteria: Meet the diagnostic requirements of the “Chinese Guidelines for the Diagnosis and Treatment of Acute Ischemic Stroke 2018” (25), and confirm that there is a new cerebral infarction by MRI, and the onset is within 2 weeks. Exclusion criteria included: ① Concomitant autoimmune diseases, tumors, blood disorders, tuberculosis, or related conditions; ② History of infectious diseases or trauma within three months; ③ Use of antibiotics, hormones, or immunosuppressive agents within three months; and ④ Incomplete information.

### 2.2 Grouping

Based on the inclusion and exclusion criteria, 416 cases of acute ischemic stroke were included as the observation group. Additionally, 60 patients admitted during the same period with dizziness/vertigo but without vascular diseases were included as the control group. There were no significant differences in baseline characteristics, including age and gender, between the observation and control groups.

To investigate the dynamic changes of peripheral blood lymphocytes at different phases after ischemic stroke, sixty cases of acute ischemic stroke patients were randomly selected. The levels of their lymphocytes were compared with those of 55 cases of transient ischemic attack patients (characterized by brief attacks that could recur and lack of imaging evidence of cerebral infarction), 21 cases of patients in the recovery phase of ischemic stroke (onset more than six months prior), and 47 cases of patients in the sequelae phase (onset more than one year prior).

### 2.3 Determination of clinical characteristics

Carotid atherosclerosis was defined as an intima-media thickness greater than or equal to 1 or the presence of plaques by carotid ultrasound. Additionally, several factors defined the risk of cerebrovascular diseases: ① Comorbidities: hypertension, diabetes, coronary heart disease, atrial fibrillation; ② Personal history: current smoking, current alcohol consumption; and ③ Medication history: antihypertensive drugs, antidiabetic drugs, statins, antiplatelet drugs. Medical history was defined as having a history, having no history but a history of medication, or being diagnosed during the current hospitalization. Current smoking was defined as smoking at least one cigarette per day for at least one year and currently reporting smoking. Current alcohol consumption was defined as consuming any type of alcoholic beverage at least once a week in the past three years.

### 2.4 Data collection

Demographic data, cerebrovascular disease risk factors, laboratory tests (lymphocyte subpopulations, lipid profiles), clinical characteristics, and carotid ultrasound results were collected. Laboratory tests were uniformly performed on the second day after admission. Venous blood was collected in the morning to measure the proportions of various lymphocyte subpopulations and lipid profiles. Lymphocyte subpopulations included T lymphocytes (CD3+), T helper/inducer lymphocytes (CD4+), T cytotoxic/suppressor lymphocytes (CD8+), natural killer (NK) lymphocytes (CD16 + CD56 + CD3-), and B lymphocytes (CD19+). Clinical characteristics included admission time, baseline systolic and diastolic blood pressure, National Institute of Health Stroke Scale (NIHSS) score at admission and on the third day, post-stroke infections, progressive stroke, and other data. Carotid ultrasound primarily assessed intima-media thickness and the presence of plaques in the left and right carotid arteries. Vascular recanalization treatment included intravenous thrombolysis or arterial thrombectomy. Functional assessments were performed using the modified Rankin Scale (mRS) at discharge and one year later to evaluate prognosis.

### 2.5 Statistical methods

Statistical analysis was conducted using SPSS 26 and GraphPad 8.0 software. Two-tailed tests were performed, and *p*-values less than 0.05 were considered statistically significant. Continuous data were presented as mean ± standard deviation or median (25th–75th percentile). The Shapiro–Wilk test was used to assess normality, and independent sample t-tests were applied for normally distributed data; otherwise, non-parametric tests were used. Count data were expressed as numbers (constituent ratios), and group comparisons were made using the chi-square test.

Single-factor logistic regression and binary logistic regression were used to analyze the correlation of atherosclerosis. B lymphocytes were grouped according to quartiles, and data were presented as odds ratios (OR) and 95% confidence intervals (CI). The Receiver Operating Characteristic (ROC) curve was used to calculate the optimal diagnostic threshold for adverse outcomes and carotid atherosclerosis.

## 3 Results

### 3.1 Clinical characteristics of acute ischemic stroke patients and dizziness/vertigo control group patients

**Figure 1:**
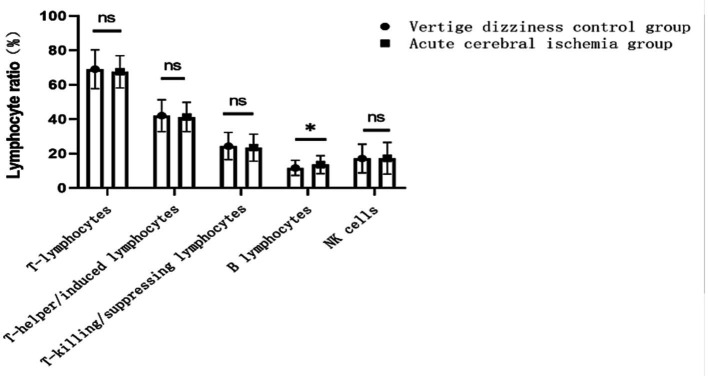
Changes in the proportion of lymphocyte subsets to controls in patients with acute ischemic stroke. ns indicates no difference, * indicates P less than 0.05 vs. control group.

Compared to the control group (patients with dizziness/vertigo), the observation group (acute ischemic stroke patients) showed no significant differences in age or gender. However, a significantly higher prevalence of hypertension, diabetes, smoking history, alcohol consumption history, and antiplatelet drug usage was observed in the observation group. Additionally, the proportion of B lymphocyte subpopulations (13.6 ± 5.3 vs. 11.7 ± 4.4%, *p* = 0.006) was significantly higher in the observation group than in the control group. There were no statistically significant differences in the proportions of other lymphocyte subpopulations, including T lymphocytes, T helper/inducer lymphocytes, T cytotoxic/suppressor lymphocytes, and NK lymphocytes (See Figure 1; Table 1). Furthermore, there were no significant differences in various lipid parameters (TG, TC, HDL-C, VLDL-C, and LDL-C).

**Table 1:** The demographic characteristics, risk factors, and clinical parameters of the study population.

	Acute ischemic stroke *n* = 416	Control *n* = 60	*p* value
Demographic information			
Age	64 ± 13.8	61 ± 14.3	0.120
Male (%)	279 (67)	38 (63)	0.566
Comorbidities *n* (%)			
Hypertension	307 (74)	35 (58)	**0.013**
Diabetes	115 (28)	7 (11)	**0.008**
Coronary heart disease	28 (7)	3 (5)	0.612
Personal history *n* (%)			
Smoking now	143 (34)	10 (17)	**0.006**
Drinking now	96 (23)	3 (5)	**0.001**
Medication history *n* (%)			
Antihypertensive drugs	245 (59)	31 (52)	0.289
Hypoglycemic drugs	94 (23)	7 (12)	0.053
Statins	53 (13)	3 (5)	0.082
Antiplatelet drugs	66 (16)	2 (3)	**0.010**
Laboratory examination (lymphatic *n* %, blood lipid mmol/L)			
T lymphocytes	67.7 ± 9.2	69.1 ± 11.3	0.255
T-helper/inducible lymphocytes	41.3 ± 8.4	42.1 ± 9.3	0.458
T-killing/suppressor lymphocytes	23.5 ± 7.9	24.4 ± 8.4	0.388
B lymphocytes	13.6 ± 5.3	11.7 ± 4.4	**0.006**
NK lymphocytes	17.1 ± 8.9	17.2 ± 8.4	0.994
TG	1.68 ± 1.54	1.76 ± 1.30	0.607
TC	4.71 ± 1.20	4.83 ± 0.95	0.425
HDL-C	1.13 ± 0.32	1.22 ± 0.43	0.054
VLDL-C	0.82 ± 0.64	0.87 ± 0.55	0.527
LDL-C	2.77 ± 0.94	2.77 ± 0.80	0.995
Clinical features			
Time of onset (h)	53 ± 61.9		
Baseline systolic blood pressure (mmHg)	150 ± 23.3		
Baseline diastolic blood pressure (mmHg)	83 ± 14.7		
Admission NIHSS score	4 (2–5)		

Bold: *P*<0.05.

### 3.2 Dynamic changes in peripheral blood lymphocyte subpopulations at different phases of cerebral ischemia

**Figure 2:**
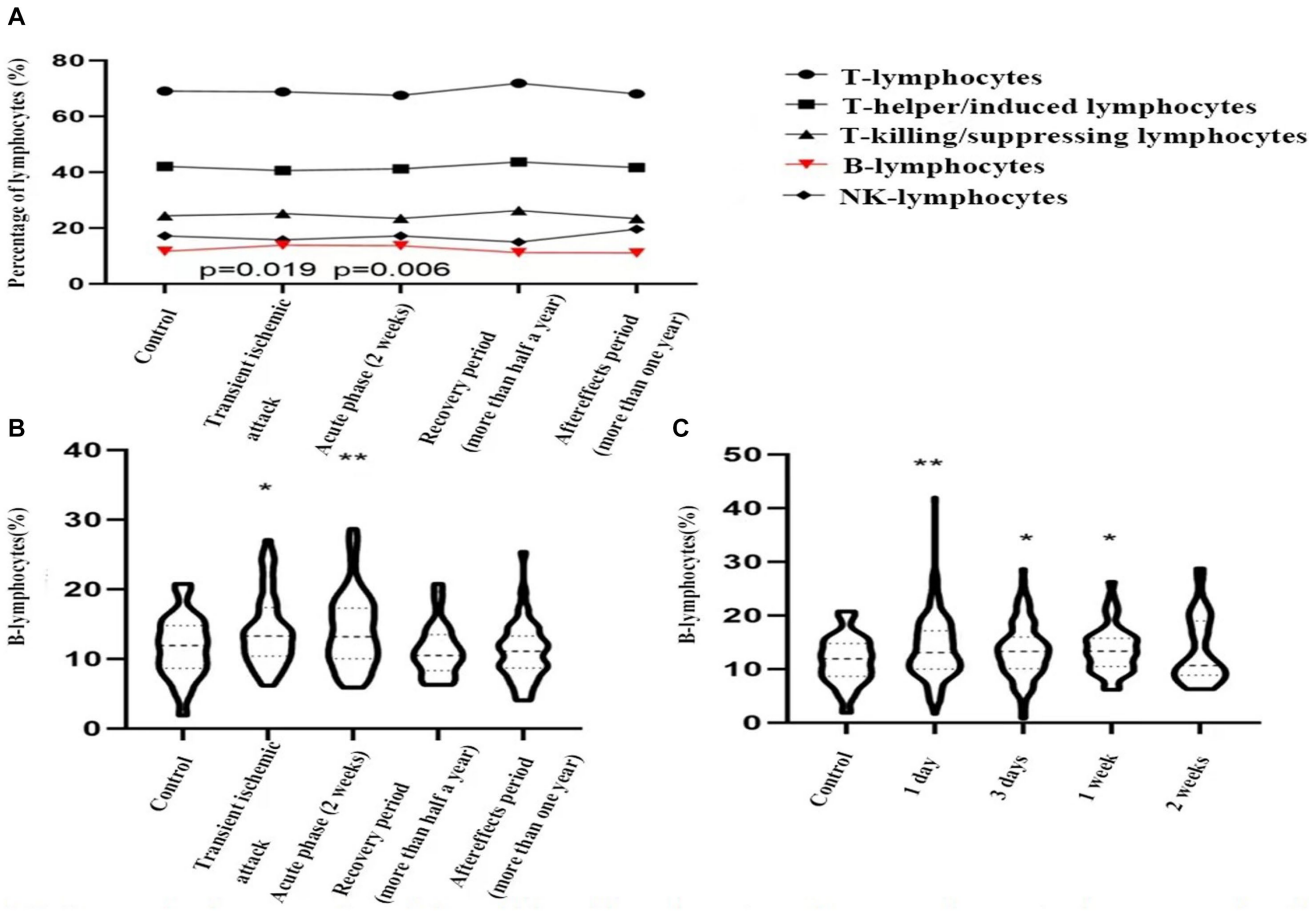
Dynamic changes of peripheral blood lymphocyte sub-groups in control group and patients with transient ischemic attack (TIA), acute ischemic stroke, stroke recovery period, stroke sequelae phase. **(A)** TIA vs. control group, *p* = 0.019; acute ischemic stroke vs. control group, *p* = 0.006; **(B,C)**. *indicates *p* < 0.05, **indicates *p* < 0.01 vs. control group.

Based on the timing of cerebral ischemic events, patients were divided into the acute phase, recovery phase, and sequelae phase. A total of 60 patients with acute ischemic strokes were randomly selected. In addition, 55 patients with transient ischemic attacks, 21 patients in the recovery phase, and 47 patients in the sequelae phase were enrolled. The proportions of various lymphocyte subpopulations in these groups were compared to a control group of 60 patients with dizziness/vertigo. No significant differences were observed in the proportions of T lymphocytes, T helper/inducer lymphocytes, T cytotoxic/suppressor lymphocytes, or NK lymphocytes compared to the control group. However, the proportion of B lymphocytes was significantly higher in both transient ischemic attack (13.9 ± 5.1% vs. 11.7 ± 4.4%, *p* = 0.019) and acute ischemic stroke (14.1 ± 5.3% vs. 11.7 ± 4.4%, *p* = 0.006) groups compared to the control group, indicating statistical significance (see Figures 2A,B).

Subsequently, acute ischemic stroke patients were grouped based on hospital admission time: one day, three days, one week, and two weeks, consisting of 232 cases, 105 cases, 44 cases, and 35 cases, respectively. These groups were compared to the control group. The proportion of B lymphocytes was significantly higher in the first day (14.7 ± 4.9% vs. 11.7 ± 4.4%, *p* = 0.006), third day (14.4 ± 4.9% vs. 11.7 ± 4.4%, *p* = 0.028), and first week (13.7 ± 4.4% vs. 11.7 ± 4.4%, *p* = 0.025) compared to the control group. However, by the second week (13.4 ± 6.2% vs. 11.7 ± 4.4%, *p* = 0.132), the proportion had decreased to baseline levels (see Figure 2C).

### 3.3 Relationship between peripheral blood B lymphocyte proportions and clinical characteristics

Based on whether the proportion of peripheral blood B lymphocytes exceeded the control group’s average of 11.7%, B lymphocytes were divided into two groups: “B^low^” and “B^high^.” Clinical characteristics between these two groups were compared (see Table 2). Patients in the B lymphocyte low proportion group differed significantly from those in the high proportion group in terms of age (67 ± 13.2 vs. 63 ± 13.9, *p* = 0.001) and gender (126 (75%) vs. 153 (61%), *p* = 0.003), indicating that younger and female ischemic stroke patients had a higher proportion of B cells. Significant differences were observed in various blood lipid indicators, with the high B lymphocyte proportion group showing higher lipid levels. Specifically, the lipid indicators in the B^low^ and B^high^ groups were as follows: TG (1.63 ± 2.04 vs. 1.7 ± 1.08 mmol/L, *p* = 0.001), TC (4.59 ± 1.34 vs. 4.79 ± 1.10 mmol/L, *p* = 0.028), HDL-C (1.18 ± 0.32 vs. 1.09 ± 0.32 mmol/L, *p* = 0.002), VLDL-C (0.81 ± 0.87 vs. 0.82 ± 0.41 mmol/L, *p* = 0.003), and LDL-C (2.61 ± 0.89 vs. 2.88 ± 0.96 mmol/L, *p* = 0.005). The proportion of carotid artery atherosclerosis in the B^high^ group was significantly higher at 41% compared to the 16% in the B^low^ group.

**Table 2:** Comparison of baseline information of lymphocytes in B^low^ and B^high^ groups.

	B^low^ *n* = 167	B^high^ *n* = 249	*p* value
Demographic information			
Age	67 ± 13.2	63 ± 13.9	**0.001**
Male (%)	126(75)	153(61)	**0.003**
Past history *n* (%)			
Stroke	40(24)	49(19)	0.297
Hypertension	131(78)	176(71)	0.078
Diabetes	45(27)	70(28)	0.794
Coronary heart disease	15(9)	13(5)	0.133
Atrial fibrillation	17(10)	23(9)	0.749
Personal History *n* (%)			
Smoking now	49(29)	94(37)	0.077
Drinking now	38(23)	58(23)	0.898
Medication history *n* (%)			
Antihypertensive drugs	104(62)	141(57)	0.251
Glucose-lowering drugs	36(22)	58(23)	0.678
Statin	26(16)	27(11)	0.156
Antiplatelet agents	28(17)	38(15)	0.680
Laboratory tests (mmol/L)			
TG	1.63 ± 2.04	1.7 ± 1.08	**0.001**
TC	4.59 ± 1.34	4.79 ± 1.10	**0.028**
HDL-C	1.18 ± 0.32	1.09 ± 0.32	**0.002**
VLDL-C	0.81 ± 0.87	0.82 ± 0.41	**0.003**
LDL-C	2.61 ± 0.89	2.88 ± 0.96	**0.005**
Clinical characteristics			
Time to onset of disease (h)	58 ± 69.6	50 ± 56.1	0.556
Baseline systolic blood pressure(mmHg)	150 ± 23.9	150 ± 23.0	0.807
Baseline diastolic blood pressure(mmHg)	82 ± 15.7	83 ± 14.1	0.480
Admission NIHSS score	4(2–6)	4(2–5)	0.428
Carotid ultrasound			
Carotid athe%	26(16)	103(41)	**<0.001**

Bold: *P*<0.05.

### 3.4 Correlation analysis between B lymphocytes and carotid atherosclerosis

**Figure 3:**
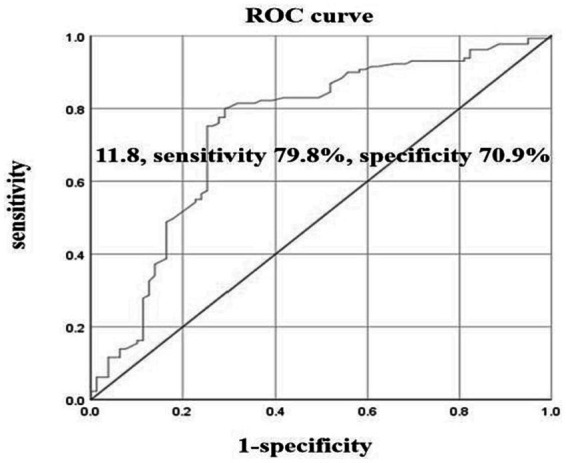
B lymphocytes predict ROC curves for carotid atherosclerosis.

Among the included acute ischemic stroke patients, 208 cases underwent carotid ultrasound. They were divided into a carotid atherosclerosis group with 149 cases and a non-carotid atherosclerosis group with 59 cases. The correlation between lymphocyte subgroups and carotid atherosclerosis was analyzed. Single-factor analysis revealed that a decrease in the proportion of T lymphocytes (66.3 ± 8.5 vs. 69.4 ± 9.0%, *p* = 0.032), a decrease in the proportion of T cytotoxic/suppressor lymphocytes (22.4 ± 7.6 vs. 25.4 ± 8.6%, *p* = 0.017), and an increase in the proportion of B lymphocytes (14.9 ± 4.6 vs. 11.0 ± 4.7%, *p* < 0.001) were associated with carotid atherosclerosis, with *p*-values of 0.032, 0.017, and less than 0.001, respectively (see Table 3). The B lymphocyte proportions were divided into four groups based on quartiles, with the first quartile as the reference. Single-factor analysis showed that the second, third, and fourth quartile groups all had significant associations, indicating that an increased proportion of B lymphocytes increased the risk of carotid atherosclerosis. Even after adjusting for confounding factors, the results remained significant. The risk of carotid atherosclerosis in the third quartile group was 7.68 times that of the first quartile group (95% confidence interval 2.98–19.79, *p* = 0.002), and the risk in the fourth quartile group was 7.71 times that of the first quartile group (95% confidence interval 2.98–19.93, *p* = 0.002) (see Table 4). The OR decreases from group III to group IV, which indicated that as the proportion of B lymphocytes increases, the risk of atherosclerosis increases. ROC curve analysis showed that the optimal cutoff value for diagnosing carotid atherosclerosis based on the B lymphocyte proportion was 11.8, with a sensitivity of 79.8%, specificity of 70.9%, and an area under the curve of 0.745 (see Figure 3).

**Table 3:** Univariate analysis of carotid atherosclerosis patients and lymphocyte subsets.

	Total	Non-atherosclerosis *n* = 59	Carotid atherosclerosis *n* = 149	*p* value
T lymphocyte	67.5 ± 8.9	69.4 ± 9.0	66.3 ± 8.5	**0.032**
T-helper/induced lymphocytes	41.1 ± 8.2	40.2 ± 9.6	41.6 ± 7.3	0.402
T kills/inhibits lymphocytes	23.6 ± 8.1	25.4 ± 8.6	22.4 ± 7.6	**0.017**
B lymphocytes	13.4 ± 5.0	11.0 ± 4.7	14.9 ± 4.6	**<0.001**
NK lymphocytes	17.7 ± 9.2	18.0 ± 9.2	17.4 ± 9.1	0.515

Bold: *P*<0.05.

**Table 4:** Relationship between different levels of B lymphocytes and carotid atherosclerosis.

	Before adjustment			* After adjustment		
B lymphocyte%	OR value	95% confidence interval	*p*-value	OR value	95% confidence interval	*p*-value
Group I (≤9.9)	1			1		
Group II (9.9–13.1)	3.44	1.54–7.69	0.003	3.2	1.40–7.34	0.006
Group III (13.1–17.0)	9.84	3.93–24.61	0.001	7.68	2.98–19.79	0.002
Group IV (>17)	9.6	3.83–24.04	0.001	7.71	2.98–19.93	0.002

*Smoking, alcohol consumption, T lymphocytes, and T killer/suppressed lymphocytes were included in the regression model.

## 4 Discussion

Circulating lymphocytes changes following ischemic stroke are associated with increased susceptibility to infection and poor patient outcome due to their role in exacerbating the ischemic injury and long-term disability. Global understanding of early changes to systemic immunity is critical to identify immune targets to improve clinical outcome. However, changes in the number of immune cells at different stage after stroke remain controversial. Some evidence revealed a rapid decline in lymphocytes and NK cells in blood early after stroke (26), while other studies showed differential responses in different immune cell populations (27–29). A possible explanation is that signs of strong immunosuppression after stroke do not have the same effect on all lymphocyte subsets, which is related to lymphocyte subset specificity (30). In this study, we observed prominent increase in B cells after stroke. One interesting finding is that the proportion of B lymphocytes is related to age and gender, with higher proportions observed in young individuals and female patients. This correlation may be linked to the more active immune system in these two demographic groups, although the underlying mechanisms require further investigation.

Different B cell subsets have been proposed on the basis of expression levels of transcription factors as well as specific surface proteins. Peripheral blood B cells can be divided into 8 continuously differentiated subsets by the expression of immunoglobulin M (IgM), Ig D, CD10, CD19, CD24, CD27 and CD38, followed by Immature B, T1 Transitional B, T2 Transitional B, T3 Transitional B, Naive B, Unswitched Memory B, and Switched Memory B, Plasmablast. Different B cells play different roles in atherosclerosis and ischemic stroke (31–33). More and more findings allude to the potential candidacy of these subpopulations as therapeutic targets in the realm of ischemic stroke prevention and management.

Atherosclerosis constitutes a major pathological mechanism underlying ischemic stroke, wherein the accumulation of oxidized lipids associated with lipid metabolism abnormalities within the vascular wall is a central process in atherosclerotic development and progression (34). Analysis of genome-wide association and transcriptomic data suggests the involvement of B cells in the formation of atherosclerosis, and the activation and proliferation of B cells are significant risk factors in the development of ischemic cerebrovascular diseases (35). This study revealed that among various lymphocyte subpopulations, elevated levels of B cell subsets in ischemic stroke patients are significantly correlated with atherosclerosis and lipid metabolism abnormalities. Patients with higher levels of B lymphocyte subsets exhibited an increased incidence of carotid artery sclerosis (41% vs. 16%), along with elevated levels of detrimental lipid parameters including triglycerides (TG), total cholesterol (TC), very low-density lipoprotein cholesterol (VLDL-C), and low-density lipoprotein cholesterol (LDL-C), accompanied by reduced levels of protective high-density lipoprotein cholesterol (HDL-C). As B lymphocyte levels rose, the occurrence and severity of atherosclerosis also increased. A higher proportion of B lymphocytes (optimal threshold at 11.8%) demonstrated elevated sensitivity (79.8%) and specificity (70.9%) in diagnosing atherosclerosis. Although the mechanism by which B cells regulate lipid metabolism and atherosclerosis remains to be investigated. These suggest that B cell subpopulations might participate in the regulation of atherosclerosis and contribute to the modulation of ischemic stroke. Thus, targeted therapies involving B cells could potentially play a significant regulatory role in both the prevention and treatment of ischemic stroke, benefiting patients.

However, animal experimentation results indicate that after receiving whole spleen B cell transplantation, mice with splenectomy and apolipoprotein E knockout (ApoE−/−) exhibited significantly reduced aortic root atherosclerotic plaques, showing a protective role of B cells in atherosclerosis. Employing univariate and multivariate Cox proportional hazard models to scrutinize the relationship between B cell subtypes, circulating antibodies, and secondary cardiovascular incidents over a 3-year follow-up period, it has been discerned that specific B cell subgroups possess inherent potential in prognosticating and preventing secondary cardiovascular events in patients afflicted by atherosclerosis (36, 37). These datas suggest that the main focus of work to find the immune intervention targets for prevention and treatment of atherosclerotic diseases should be to find the main pathogenic B-cell subsets.

Following an ischemic stroke event, B cells exhibit a sustained presence within cerebral tissues. In murine models, B cells have been identified in the brain up to 10 weeks post-stroke, while human ischemic stroke patients continue to exhibit elevated peripheral B cell levels beyond the 12-week mark post-ischemia. Furthermore, the synthesis of immunoglobulins in the cerebrospinal fluid of stroke patients persists for several months post-ischemia (38). Our study also found an increase in peripheral B cells after stroke, which increased continuously for at least 7 days, decreased to baseline levels by two weeks. Unfortunately, because this study was retrospective, the dynamic changes of lymphocytes in each patient could not be observed. Future prospective studies are expected to fill this gap.

Different B cell subpopulations exhibit distinct regulatory functions at various stages of the post-stroke period. On one hand, B cells are a major source of brain-derived neurotrophic factor (BDNF), and their neurotrophic capabilities penetrate the post-stroke brain, inducing early antigen-independent protection against ischemic injury, participate in the restoration of neural plasticity in cerebral motor and cognitive regions, serving as a defense mechanism against potential recurrent immune injuries (20). On the other hand, they can adversely affect the hippocampus by generating antibodies, activating the complement system, leading to delayed cognitive impairments, and potentially infiltrating neighboring unaffected healthy tissues, thereby exacerbating the pathological condition (23, 24).

Over the years, many scholars have done a lot of work in trying to discover disease-causing B cell subsets. CD19 + CD86 + B cells are associated with pro-inflammatory factor release, carotid artery stenosis, and high risk of stroke. CD19 + CD40+ B cells are associated with a low risk of stroke (39). Depletion of B2 cells with monoclonal antibody against CD20 or BAFF receptor or BAFF receptor-deficient mice improves atherosclerosis. B2 cells can promote atherosclerosis by producing IgG, secreting pro-inflammatory cytokines, and activating CD4 T cells (15, 16). CD11 b^high^ B cells regulate microglia phenotype and increase microglia phagocytosis in both *ex vivo* and *in vivo* settings, likely by production of regulatory cytokines (e.g., TNF-α). As both APCs and adaptive immune cells with long-term memory function, B cells are uniquely positioned to regulate acute and chronic phases of the post-stroke immune response, and their influence is subset specific (40). B cells producing IL-10 and Treg cells exert their influence by modulating neutrophils, thereby mitigating inflammatory responses and reducing infarct size (41). Notably, memory B cells have demonstrated a positive correlation with improved postoperative outcomes in patients undergoing carotid endarterectomy (42).

In conclusion, B cell-targeted therapy emerges as a promising and complementary approach for the treatment of ischemic stroke. Its potential benefits lie in its ability to extend the therapeutic window, minimize hemorrhagic complications, and its relevance across the pre-onset, acute, and chronic phases of the disease. However, it’s essential to recognize that different subpopulations of B cells exert distinct regulatory functions during various stages of ischemic stroke, driven by the dynamic changes in the immune milieu. This study, being retrospective and single-center with a relatively modest sample size, presents certain limitations. Furthermore, it lacks the dynamic tracking of lymphocyte subpopulation alterations in individual patients. Future research should focus on expanding the sample size and conducting comprehensive, systematic investigations into the dynamics of B cell subpopulations following ischemic stroke. This will entail gaining deeper insights into the dynamic changes in B lymphocyte subpopulations in acute ischemic stroke patients, conducting in-depth exploration of the roles played by diverse B cell subpopulations at different stages of ischemic stroke, and delving into the identification of key subgroups and core mechanisms through which B cells regulate ischemic stroke. In terms of intervention assessment, it is imperative to consider both short-term and long-term outcomes, including cognitive function evaluations. Ultimately, if specific B cell subpopulations that play pivotal pathogenic roles in post-ischemic brain tissue damage can be pinpointed in clinical practice, and if targeted interventions are demonstrated to benefit stroke patients, this research may provide pivotal guidance for the clinical translation of immunomodulatory interventions as therapeutic targets in ischemic stroke.

## Data availability statement

The original contributions presented in the study are included in the article/supplementary material, further inquiries can be directed to the corresponding author.

## Ethics statement

The studies involving humans were approved by Ethics Committee of the Second Affiliated Hospital of Soochow University. The studies were conducted in accordance with the local legislation and institutional requirements. The ethics committee/institutional review board waived the requirement of written informed consent for participation from the participants or the participants’ legal guardians/next of kin because this study is a retrospective clinical study, which does not involve any prospective observation or intervention, and the information obtained is only used for the study protocol to effectively protect the privacy of the subjects.

## Author contributions

YuhZ: Conceptualization, Investigation, Software, Writing – original draft. YJ: Conceptualization, Data curation, Formal analysis, Software, Validation, Writing – original draft, Writing – review & editing. YutZ: Conceptualization, Data curation, Investigation, Software, Validation, Writing – original draft. YF: Conceptualization, Data curation, Validation, Visualization, Investigation, Software, Writing – review & editing. PF: Conceptualization, Data curation, Investigation, Validation, Writing – review & editing. XF: Data curation, Investigation, Validation, Writing – review & editing. KL: Data curation, Investigation, Software, Validation, Writing – review & editing. JZ: Conceptualization, Data curation, Investigation, Software, Validation, Writing – review & editing. YD: Conceptualization, Formal analysis, Resources, Software, Validation, Writing – review & editing. SY: Writing – review & editing, Data curation, Formal analysis, Validation. YanZ: Conceptualization, Data curation, Formal analysis, Funding acquisition, Investigation, Methodology, Project administration, Resources, Supervision, Validation, Visualization, Writing – original draft, Writing – review & editing.
